# Diversity of Intestinal *Clostridium coccoides* Group in the Japanese Population, as Demonstrated by Reverse Transcription-Quantitative PCR

**DOI:** 10.1371/journal.pone.0126226

**Published:** 2015-05-22

**Authors:** Takashi Kurakawa, Kiyohito Ogata, Kazunori Matsuda, Hirokazu Tsuji, Hiroyuki Kubota, Toshihiko Takada, Yukiko Kado, Takashi Asahara, Takuya Takahashi, Koji Nomoto

**Affiliations:** 1 Yakult Central Institute, 5–11 Izumi, Kunitachi, Tokyo, 186–8650, Japan; 2 Yakult Honsha European Research Center for Microbiology, ESV, Technologiepark 4, Gent-Zwijnaarde, 9052, Belgium; Institute Pasteur, FRANCE

## Abstract

We used sensitive rRNA-targeted reverse transcription-quantitative PCR (RT-qPCR) to quantify the *Clostridium coccoides* group, which is a major anaerobic population in the human intestine. For this purpose, the *C*. *coccoides* group was classified into 3 subgroups and 19 species for expediency in accordance with the existing database, and specific primers were newly developed to evaluate them. Population levels of the *C*. *coccoides* group in human feces determined by RT-qPCR were equivalent to those determined by fluorescence *in situ* hybridization. RT-qPCR analysis of fecal samples from 96 volunteers (32 young children, 32 adults and 32 elderly) by using the 22 new primer sets together with the *C*. *coccoides* group-specific primer setm revealed that (i) total counts obtained as the sum of the 3 subgroups and 19 species were equivalent to the results obtained by using the *C*. *coccoides* group-specific primer set; (ii) total *C*. *coccoides*-group counts in the elderly were significantly lower than those in young children and adults; (iii) genus *Blautia* was the most common subgroup in the human intestinal *C*. *coccoides*-group populations at all age populations tested; (iv) the prevalences of *Fusicatenibacter saccharivorans* and genus *Dorea* were significantly higher in adults than in young children and the elderly; and (v) the prevalences of *C*. *scindens* and *C*. *hylemonae*, both of which produce secondary bile acid in the human intestine, were significantly higher in the elderly than in young children and adults. Hierarchical clustering and principal component analysis showed clear separation of the bacterial components between adult and elderly populations. Taken together, these data suggest that aging plays an important role in the diversity of *C*. *coccoides*-group populations in human intestinal microbiota; changes in this diversity likely influence the health of the host.

## Introduction

The human intestine is inhabited by more than 1000 bacterial species, with a total of 10^11^ to 10^12^ bacterial cells per gram of feces [1]. Among a variety of human intestinal bacteria, the strictly anaerobic *Clostridium coccoides* group constitutes 25% to 60% of the total and is thus the most dominant bacterial group [2–5]. A large number of species of genera such as *Clostridium*, *Blautia*, *Dorea*, *Eubacterium*, *Ruminococcus*, *Anaerostipes*, *Roseburia*, and *Coprococcus* belong to the group [2,5]. A clone library analysis has shown that not only culturable species but also many as-yet-uncultured bacteria are included in this group [6]: our team has recently identified a new species, *Fusicatenibacter saccharivorans*, in the group [7].

The *C*. *coccoides*-group bacteria have been reported to affect their hosts’ intestines in several ways. For example, *Eubacterium rectale*, *Eubacterium hallii*, *Eubacterium ramulus*, *Roseburia intestinalis*, and *Anaerostipes caccae* are known to produce butyrate [8,9]. *Clostridium scindens* and *Clostridium hylemonae* have high levels of bile acid 7α-dehydroxylating activity; this yields secondary bile acids such as deoxycholic acid and lithocholic acid [10]. Moreover, certain commensal *C*. *coccoides*-group species induce regulatory T-cell production in the mouse colon, suggesting that these bacteria play critical roles in immune homeostasis [11,12]. Taken together, these findings show that *C*. *coccoides* group play important roles in immunology, nutrition, and pathological processes, and hence in the health of their hosts. Despite their predominance in the human intestine and their physiological importance to the host, the composition of *C*. *coccoides* group-species in the human intestine remains unclear. YIF-SCAN (Yakult Intestinal Flora-SCAN), a highly sensitive and rapid system that uses reverse transcription-quantitative PCR (RT-qPCR), has been developed to quantify a variety of bacterial populations in the intestinal microbiota [13–17]. The sensitivity of this technique has been shown to be 100 to 1000 times higher than that of qPCR, because the rRNA copy number per cell (approximately 10^4^ copies per actively growing cell) is higher than that of rRNA genes (approximately 10 copies in a genome) [13,14,16]. Here, we developed specific primer sets for the 3 subgroups and 19 species in the *C*. *coccoides* group. We then analyzed the intestinal *C*. *coccoide-*group populations in 96 Japanese volunteers (32 young children, 32 adults, and 32 elderly) by using RT-qPCR to evaluate bacterial population diversity in the different age groups.

## Materials and Methods

### Strains and culture conditions

The strains listed in Table 1 were used. Thirty-eight strains were cultured anaerobically at 37°C in Modified GAM broth (Nissui Pharmaceutical Co., Ltd, Tokyo, Japan) containing 1.0% (wt/vol) glucose (Beckton Dickinson Co., Sparks, MD) for the following periods of time for harvest them in the early stationary phase: *A*. *caccae*, *Dorea formicigenerans*, *Eu*. *ramulus*, and *Blautia producta* for 18 h; *Clostridium hathewayi*, *Clostridium symbiosum*, *F*. *saccharivorans*, *Ruminococcus gnavus*, *Ruminococcus lactaris*, *Eu*. *rectale*, and *Eubacterium ventriosum* for 20 h; *Clostridium nexile* and *Eu*. *hallii* for 22 h; *Clostridium indolis*, *Clostridium oroticum*, *Eubacterium eligens*, *Clostridium celerecrescens*, *Clostridium sphenoides*, *Blautia hydrogenotrophica*, *Blautia schinkii*, *Ruminococcus obeum*, *Blautia coccoides*, *Blautia hansenii*, *Blautia luti*, *C*. *hylemonae*, *C*. *scindens*, *Ruminococcus torques*, *Coprococcus eutactus*, *Coprococcus comes*, *Roseburia intestinalis*, *Clostridium asparagiforme*, *Bacteroides vulgatus*, *Bifidobacterium longum*, *Collinsella aerofaciens*, *Prevotella melaninogenica*, *Clostridium perfringens*, and *Clostridium difficile* for 24 h; and *Faecalibacterium prausnitzii* for 72 h. *Lactobacillus acidophilus* was cultured anaerobically at 37°C for 24 h in Lactobacilli MRS broth (Becton Dickinson Co.). *Escherichia coli*, *Enterococcus faecalis*, and *Staphylococcus aureus* were cultured aerobically at 37°C for 16 h in Brain Heart Infusion broth (Beckton Dickinson Co.). *Campylobacter jejuni* was cultured under micro-aerophilic conditions at 37°C for 16 h in Preston broth, which contained Bacto peptone (1.0%, wt/vol; Difco Laboratories, Detroit, MI), Lab-Lemco powder (1.0%, wt/vol; Oxoid Co., Basingstoke, UK), PBS(-) (1.0%, wt/vol; Nissui Pharmaceutical Co., Ltd), sodium pyruvate (0.025%, wt/vol; Kanto Chemical Co., Tokyo, Japan), sodium disulfite (0.025%, wt/vol; Kanto Chemical Co.), and iron(III) sulfate *n*-hydrate (0.025%, wt/vol; Kanto Chemical Co.).

**Table 1:** Primer specificity.

Strain			Reaction with the following primers																					
			s-Event-F/R	s-Ehal-F/R	s-Ceut-F/R	s-Eeli-F/R	s-Erec-F/R	s-Eram-F/R	g-Blau-F/R	s-Csym-F/R	s-Fsac-F/R	s-Casp-F/R	s-Chath-F/R	sg-Cind-F/R	s-Rgna-F/R	s-Acac-F/R	s-Rint-F/R	s-Ccom-F/R	s-Cnex-F/R	g-Dor-F/R	s-Csci-F/R	s-Chyl-F/R	s-Rtor-F/R	s-Rlac-F/R
*Clostridium coccoides* group																								
	*Eubacterium ventriosum*	ATCC 27560^T^	+	-	-	-	-	-	±	±	-	-	-	-	-	-	-	-	-	-	-	-	-	-
	*Eubacterium hallii*	DSM 3353^T^	-	+	-	-	-	-	-	-	-	-	-	-	-	-	-	-	-	-	-	-	-	-
	*Coprococcus eutactus*	ATCC 27759^T^	-	-	+	-	-	-	-	-	-	-	-	-	-	-	-	-	-	-	-	-	-	-
	*Eubacterium eligens*	DSM 3376^T^	-	-	-	+	-	-	-	-	-	-	-	-	-	-	-	-	-	-	-	-	-	-
	*Eubacterium rectale*	ATCC 33656^T^	-	-	-	-	+	-	-	±	-	-	-	-	-	-	-	-	-	-	-	-	-	-
	*Eubacterium ramulus*	ATCC 29099^T^	-	-	-	-	-	+	-	±	-	-	-	-	-	-	-	-	-	-	-	-	-	-
	*Blautia hydrogenotrophica*	DSM 10507^T^	-	-	-	-	-	-	+	-	-	-	-	-	-	-	-	-	-	-	-	-	-	-
	*Blautia luti*	DSM 14534^T^	-	-	-	-	-	-	+	-	-	-	-	-	-	-	-	-	-	-	-	-	-	-
	*Ruminococcus obeum*	ATCC 29174^T^	-	-	-	-	-	-	+	-	-	-	-	-	-	-	-	-	-	-	-	-	-	-
	*Blautia schinkii*	DSM 10518^T^	-	-	-	-	-	-	+	-	-	-	-	-	-	-	-	-	-	-	-	-	-	-
	*Blautia hansenii*	ATCC 27752^T^	-	-	-	-	-	-	+	-	-	-	-	-	-	-	-	-	-	-	-	-	-	-
	*Blautia producta*	JCM 1471^T^	-	-	-	-	-	-	+	-	-	-	-	-	-	-	-	-	-	-	-	-	-	-
	*Blautia coccoides*	JCM 1395^T^	-	-	-	-	-	-	+	-	-	-	-	-	-	-	-	-	-	-	-	-	-	-
	*Clostridium symbiosum*	JCM 1297^T^	-	-	-	-	-	-	±	+	-	-	-	-	-	-	-	-	-	-	-	-	-	-
	*Fusicatenibacter saccharivorans*	JCM 18507^T^	-	-	-	-	-	-	-	-	+	-	-	-	-	-	-	-	-	-	-	-	-	-
	*Clostridium asparagiforme*	DSM 15981^T^	-	-	-	-	-	-	-	±	-	+	-	-	-	-	-	-	-	-	-	-	-	-
	*Clostridium hathewayi*	DSM 13479^T^	-	-	-	-	-	-	-	-	-	-	+	-	-	-	-	-	-	-	-	-	-	-
	*Clostridium indolis*	JCM 1380^T^	-	-	-	-	-	-	-	±	-	-	-	+	-	-	-	-	-	-	-	-	-	-
	*Clostridium celerecrescens*	DSM 5628^T^	-	-	-	-	-	-	±	-	-	-	-	+	-	-	-	-	-	-	-	-	-	-
	*Clostridium sphenoides*	JCM 1415^T^	-	-	-	-	-	-	±	-	-	-	-	+	-	-	-	-	-	-	-	-	-	-
	*Ruminococcus gnavus*	ATCC 29149^T^	-	-	-	-	-	-	-	±	-	-	-	-	+	-	-	-	-	-	-	-	-	-
	*Anaerostipes caccae*	DSM 14662^T^	-	-	-	-	-	-	-	-	-	±	-	-	-	+	-	-	-	-	-	-	-	-
	*Roseburia intestinalis*	DSM 14610^T^	-	-	-	-	-	-	-	-	-	-	-	-	-	-	+	-	-	-	-	-	-	-
	*Coprococcus comes*	ATCC 27758^T^	-	-	-	-	-	-	-	-	-	-	-	-	-	-	-	+	-	-	-	-	-	-
	*Clostridium nexile*	ATCC 27757^T^	-	-	-	-	-	-	-	-	-	-	-	-	±	-	-	-	+	-	-	-	-	-
	*Dorea formicigenerans*	DSM 3992^T^	-	-	-	-	-	-	-	-	-	-	-	-	-	-	-	-	-	+	-	-	-	-
	*Clostridium scindens*	JCM 6567^T^	-	-	-	-	-	-	±	-	-	-	-	-	-	-	-	-	-	-	+	-	-	-
	*Clostridium hylemonae*	DSM 15053^T^	-	-	-	-	-	-	-	-	-	-	-	-	-	-	-	-	-	-	-	+	-	-
	*Ruminococcus torques*	ATCC 17756^T^	-	-	-	-	-	-	-	-	-	-	-	-	-	-	-	-	-	-	-	-	+	-
	*Ruminococcus lactaris*	ATCC 19176^T^	-	-	-	-	-	-	-	-	-	-	-	-	-	-	-	-	-	-	-	-	-	+
	*Clostridium oroticum*	JCM 1429^T^	-	-	-	-	-	-	±	-	-	-	-	-	-	-	-	-	-	-	-	-	-	-
Other groups	* *																							
	*Faecalibacterium prausnitzii*	ATCC 27768^T^	-	-	-	-	-	-	-	-	-	-	-	-	-	-	-	-	-	-	-	-	-	-
	*Bacteroides vulgatus*	JCM 5824^T^	-	-	-	-	-	-	-	-	-	-	-	-	-	-	-	-	-	-	-	-	-	-
	*Bifidobacterium longum*	ATCC 15707^T^	-	-	-	-	-	-	-	-	-	-	-	-	-	-	-	-	-	-	-	-	-	-
	*Collinsella aerofaciens*	ATCC 25986^T^	-	-	-	-	-	-	-	-	-	-	-	-	-	-	-	-	-	-	-	-	-	-
	*Prevotella melaninogenica*	ATCC 25845^T^	-	-	-	-	-	-	-	-	-	-	-	-	-	-	-	-	-	-	-	-	-	-
	*Clostridium perfringens*	JCM 1290^T^	-	-	-	-	-	-	-	-	-	-	-	-	-	-	-	-	-	-	-	-	-	-
	*Lactobacillus acidophilus*	ATCC 4356^T^	-	-	-	-	-	-	-	-	-	-	-	-	-	-	-	-	-	-	-	-	-	-
	*Escherichia coli*	ATCC 11775^T^	-	-	-	-	-	-	-	-	-	-	-	-	-	-	-	-	-	-	-	-	-	-
	*Enterococcus faecalis*	ATCC 19433^T^	-	-	-	-	-	-	-	-	-	-	-	-	-	-	-	-	-	-	-	-	-	-
	*Staphylococcus aureus*	ATCC 12600^T^	-	-	-	-	-	-	-	-	-	-	-	-	-	-	-	-	-	-	-	-	-	-
	*Clostridium difficile*	DSM 1296^T^	-	-	-	-	-	-	-	-	-	-	-	-	-	-	-	-	-	-	-	-	-	-
	*Campylobacter jejuni*	ATCC 33560^T^	-	-	-	-	-	-	-	-	-	-	-	-	-	-	-	-	-	-	-	-	-	-

### Total RNA isolation from bacterial culture

For RNA stabilization, 200 μl of RNA*later* (Ambion Inc., Austin, TX) was added to each fresh bacterial culture (100 μl). After being kept for 10 min at room temperature, the bacterial suspensions were centrifuged at 4°C at 13,000*g* for 10 min. Pellets were stored at -80°C until used for RNA extraction. RNA extraction was performed by using a method described previously [16]. Briefly, the thawed sample was resuspended in a solution containing 346.5 μl of RLT buffer (Qiagen Sciences, Germantown, MD), 3.5 μl of β-mercaptoethanol (Sigma-Aldrich Co., St. Louis, MO) and 100 μl of Tris-EDTA buffer (Wako Pure Chemical Industries, Ltd., Osaka, Japan). Glass beads (300 mg; diameter, 0.1 mm) (BioSpec Products, Inc., Bartlesville, OK) were added to the suspension, and the mixture was vortexed vigorously for 5 min with a ShakeMaster Auto (BioMedical Science Inc., Tokyo, Japan). Then 500 μl of water-saturated phenol (Wako Pure Chemical Industries, Ltd.) was added to the mixture, which was then incubated at 60°C for 10 min. After the incubation, 100 μl of chloroform-isoamylalcohol (24:1) was added to the mixture. After centrifugation of the mixture at 13,000*g* at 4°C for 10 min, 470 μl of supernatant was collected and an equal volume of chloroform-isoamylalcohol was added to the supernatant. After centrifugation at 4°C at 12,000*g* for 5 min, 400 μl of supernatant was collected and subjected to isopropanol precipitation. Finally, the nucleic acid fraction from the bacterial culture was suspended in 100 μl of nuclease-free water (Ambion Inc.).

### Fecal collection and processing

Feces from 8 healthy Japanese adults (average age 39±8 years) were used for comparison of bacterial counts by using RT-qPCR, qPCR, and fluorescence *in situ* hybridization (FISH). Feces from 32 healthy young Japanese children (average age 3.2±0.1 years), 32 healthy adults (average age 39±11 years), and 32 healthy elderly (average age 82±6 years) were used to analyze the intestinal microbiota among different age generations by using RT-qPCR.

A spoonful of feces (0.5 g) was collected into a tube containing 2 ml of RNA*later* for nucleic acid extraction; another spoonful was collected into an empty tube for FISH analysis. Both collections were made immediately after defecation. Bacterial rRNA in feces suspended in RNA*later* solution can be kept stable during processing and storing [14].

Each fecal sample for nucleic acid analysis was weighed and suspended in 9 volumes of RNA*later* to make a fecal homogenate (100 mg feces/ml). In preparation for RNA extraction, 1 ml of PBS(-) was added to 200 μl of fecal homogenate. The fecal homogenate was centrifuged at 4°C at 13,000*g* for 10 min and all the supernatant was discarded. The precipitating pellets were stored at -80°C until used for RNA extraction. RNA extraction was performed as described above except that the final suspension volume of nuclease-free water was 1 ml. In preparation for DNA extraction, 1 ml of PBS(-) was added to 200 μl of fecal homogenate. The fecal homogenate was centrifuged at 13,000*g* for 10 min and 1 ml of the supernatant was discarded. After another wash with 1 ml of PBS(-), the pellets were stored at -30°C until used for DNA extraction. DNA extraction was performed according to the method described by Matsuki *et al*. [18].

Each fecal sample for FISH analysis was weighed and suspended in 9 volumes of PBS(-) to make a fecal homogenate (100 mg feces/ml). One hundred microliters of fecal homogenate was fixed with 300 μl of 4% paraformaldehyde at 4°C for 16 h. After fixation, 10 μl of the diluted suspension was used in the FISH analysis.

In accordance with the Declaration of Helsinki, all subjects were adequately informed of the study and provided their written informed consent to participate. In the case of minors, their guardians were also adequately informed of the study and provided written informed consent for participation. The ethics committee of Yakult Central Institute approved the study.

### Design of rRNA-targeted specific primers

**Fig 1:**
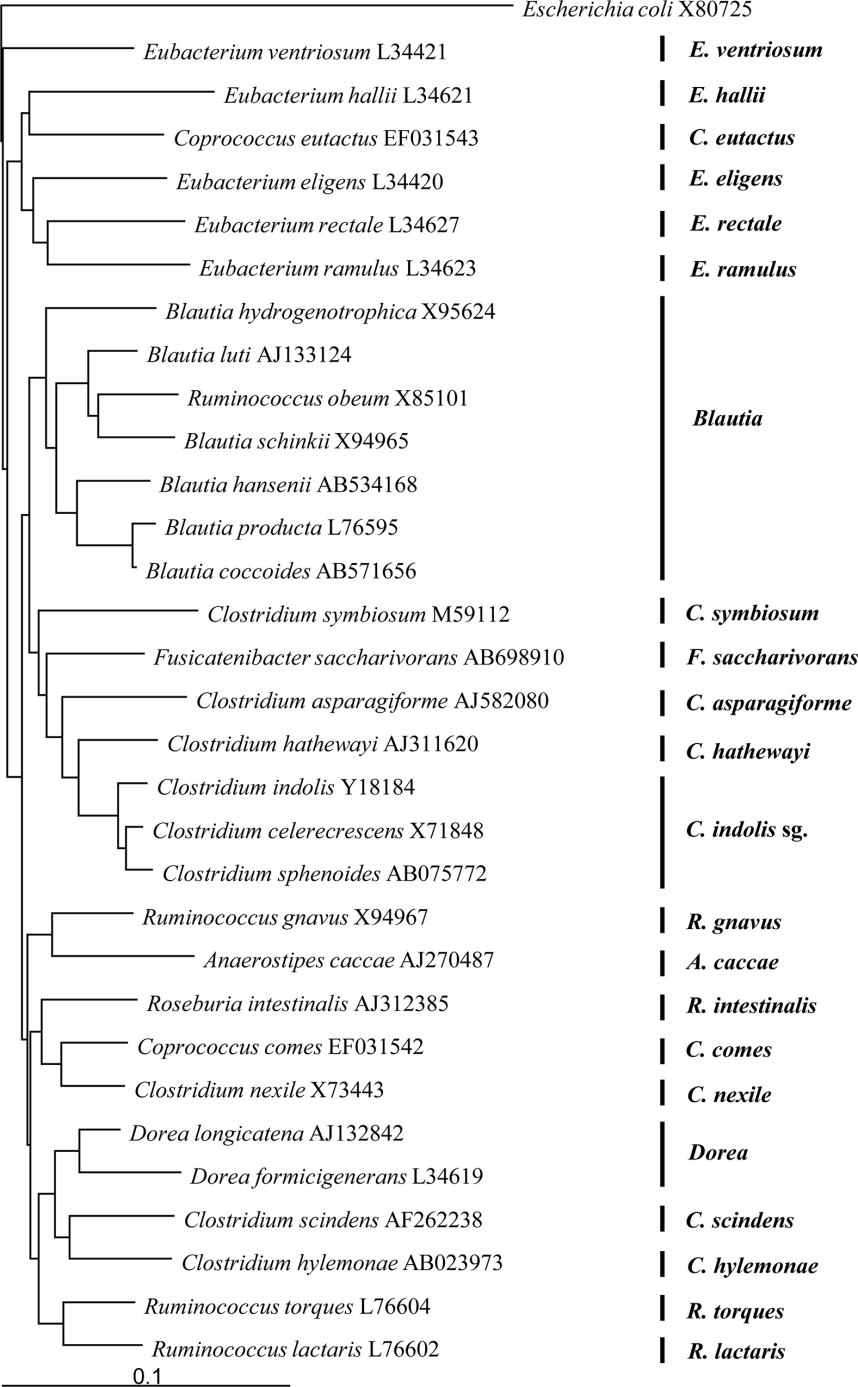
Phylogenetic tree showing the relationships among 16S rRNA gene sequences of the *Clostridium coccoides* group. Scale bar represents 0.1 substitutions per nucleotide position. The *Escherichia coli* sequence was used as an outgroup for rooting the tree.

By using 16S rRNA sequences obtained from the DDBJ/GenBank/EMBL databases, we used the program Clustal X [19] to construct multiple alignments of 31 *C*. *coccoides*-group species known to be commensals in the human intestine [1,5,6] and also of the reference organism (*E*. *coli*). We constructed a phylogenetic tree by using the neighbor-joining method with Tree View software. We then used the phylogenetic tree to classify the commensal *C*. *coccoides*-group species in the human intestinal microbiota for expediency into 3 subgroups (designated genus *Blautia*, *C*. *indolis* subgroup, and genus *Dorea*) and 19 species (*Eu*. *ventriosum*, *Eu*. *hallii*, *Co*. *eutactus*, *Eu*. *eligens*, *Eu*. *rectale*, *Eu*. *ramulus*, *C*. *symbiosum*, *F*. *saccharivorans*, *C*. *asparagiforme*, *C*. *hathewayi*, *R*. *gnavus*, *A*. *caccae*, *R*. *intestinalis*, *Co*. *comes*, *C*. *nexile*, *C*. *scindens*, *C*. *hylemonae*, *R*. *torques*, *and R*. *lactaris*) (Fig 1). After comparison of the sequences, potential target sites for specific detection were identified and 22 primer sets specific for the subgroups and species were newly constructed (Table 2). Their specificity was checked by submitting the sequences to the Probe Match program of the Ribosomal Database Project (http://rdp.cme.msu.edu/).

**Table 2:** Primer information.

group/species	Standard Strain	Strain No.	Primer name	Sequence (5'-3')	Anealing Temp (°C)	Product size (bp)
*Clostridium coccoides* group	*Blautia producta*	JCM 1471^T^	g-Ccoc-F	AAATGACGGTACCTGACTAA	55	438
			g-Ccoc-R	CTTTGAGTTTCATTCTTGCGAA		
*Eubacterium ventriosum*	*Eubacterium ventriosum*	ATCC 27560^T^	s-Event-F	GTCGGGGGACAATAGTTCC	55	451
			s-Event-R	ATTTGCTTACCCTCACGGGG		
*Eubacterium hallii*	*Eubacterium hallii*	DSM 3353^T^	s-Ehal-F	GTGTCGGGGCCGTATAGG	55	436
			s-Ehal-R	GTTCGCCTCACTCTGTGAC		
*Coprococcus eutactus*	*Coprococcus eutactus*	ATCC 27759^T^	s-Ceut-F	CTGGAGCTTGCTCCGGCCGATTT	55	655
			s-Ceut-R	GTCAGTAGCAGTCCAGTAAGT		
*Eubacterium eligens*	*Eubacterium eligens*	DSM 3376^T^	s-Eeli-F	TGTCGGGGCCCATAAGGG	55	190
			s-Eeli-R	CATTACTGTCCGGTCAGTG		
*Eubacterium rectale*	*Eubacterium rectale*	ATCC 33656^T^	s-Erec-F	TTCTGACCGGTACTTAACCGTACC	55	281
			s-Erec-R	TTTGCTCGGCTTCACAGCTTT		
*Eubacterium ramulus*	*Eubacterium ramulus*	ATCC 29099^T^	s-Eram-F	GAGCGTAGGCGGTCCTGC	55	452
			s-Eram-R	GGGAAAACACATTACATGTTCTG		
Genus *Blautia*	*Blautia producta*	JCM 1471^T^	g-Blau-F	GTGAAGGAAGAAGTATCTCGG	55	559
			g-Blau-R	TTGGTAAGGTTCTTCGCGTT		
*Clostridium symbiosum*	*Clostridium symbiosum*	JCM 1297^T^	s-Csym-F	TAAGCGCACAGTATTGCATGATA	55	815
			s-Csym-R	CGTTACTCCCCCGTCGAG		
*Fusicatenibacter saccharivorans*	*Fusicatenibacter saccharivorans*	JCM 18507^T^	s-Fsac-F	CTGCATTGGAAACTGTCTGG	55	389
			s-Fsac-R	CGTTACGGGCCGGTCATC		
*Clostridium asparagiforme*	*Clostridium asparagiforme*	DSM 15981^T^	s-Casp-F	GTTTTCGGATGGATTCTAGATG	55	568
			s-Casp-R	CTCCTGCACTCTAGCTTGA		
*Clostridium hathewayi*	*Clostridium hathewayi*	DSM 13479^T^	s-Chath-F	CTTGACATCCCACTGAAAACAC	55	162
			s-Chath-R	AGAGTGCCCGACTCTACTC		
*Clostridium indolis* subgroup	*Clostridium indolis*	JCM 1380^T^	sg-Cind-F	ACCAAGTCTTGACATCGGAATGA	55	276
			sg-Cind-R	TTGCTCCAGATCGCTCCTT		
*Ruminococcus gnavus*	*Ruminococcus gnavus*	ATCC 29149^T^	s-Rgna-F	CTTGCTGGACGATGACTGAC	55	269
			s-Rgna-R	CTCCGATTAAAGAGCGGTCAGA		
*Anaerostipes caccae*	*Anaerostipes caccae*	DSM 14662^T^	s-Acac-F	GTTTTCGGATGGATTTCCTATAT	55	121
			s-Acac-R	CTTTTCACACTGAATCATGCGATT		
*Roseburia intestinalis*	*Roseburia intestinalis*	DSM 14610^T^	s-Rint-F	GCACAGGGTCGCATGACCT	60	818
			s-Rint-R	AACACATTACATGTTCTGTCATC		
*Coprococcus comes*	*Coprococcus comes*	ATCC 27758^T^	s-Ccom-F	GTGACCGGCGTGTAATGACG	55	145
			s-Ccom-R	CAGAGTGCCCATCCGAATTG		
*Clostridium nexile*	*Clostridium nexile*	ATCC 27757^T^	s-Cnex-F	GGATTTCTTCGGATTGAAGTTTTT	55	517
			s-Cnex-R	TTTCACATCAGACTTACACAAC		
Genus *Dorea*	*Dorea formicigenerans*	DSM 3992^T^	g-Dor-F	GCAGCTAACGCAATAAGCAG	55	155
			g-Dor-R	CTTCCATTACGAAGCGGTC		
*Clostridium scindens*	*Clostridium scindens*	JCM 6567^T^	s-Csci-F	GCATTTGGAACTGCGTGG	55	587
			s-Csci-R	CGTTACGCGCTTTGGCATCG		
*Clostridium hylemonae*	*Clostridium hylemonae*	DSM 15053^T^	s-Chyl-F	AAGAGATTAGCTTGCTAAGATCAG	55	141
			s-Chyl-R	TCTACCATGCGGTACTGAGGT		
*Ruminococcus torques*	*Ruminococcus torques*	ATCC 17756^T^	s-Rtor-F	CGAAGCACTTTGCTTAGA	55	526
			s-Rtor-R	ACATCAGACTTGCCCATC		
*Ruminococcus lactaris*	*Ruminococcus lactaris*	ATCC 19176^T^	s-Rlac-F	GGGAGCGTAGACGGAGCA	55	452
			s-Rlac-R	AAGCAGACATTACTCTGCCG		

### RT-qPCR

RT-qPCR was performed with a Qiagen OneStep RT-PCR kit (Qiagen GmbH, Hilden, Germany). Each reaction mixture (10 μl) was composed of 1× Qiagen OneStep RT-PCR buffer, 0.5× Q-solution buffer, each deoxynucleoside triphosphate at a concentration of 400 μM, a 1:100,000 dilution of SYBR green I (BioWhittaker Molecular Applications, Rockland, ME), 0.4 μl of Qiagen OneStep RT-PCR enzyme mixture, and 5 μl of template RNA. Each primer set was added at a concentration of 0.6 μM in accordance with the method used in previous reports [14,16], except in the case of s-Acac-F/R, which was added at a concentration of 2.1 μM. The reason for using a different concentration of the s-Acac-F/R primer set in the reaction mixture was that RT-PCR efficiency was quite low when s-Acac-F/R was used at a concentration of 0.6 μM or 0.12 μM, but it improved substantially at a concentration of 2.1 μM (data not shown). The reaction mixture was incubated at 50°C for 30 min for reverse transcription. The continuous amplification program consisted of one cycle at 95°C for 15 min, followed by 45 cycles at 94°C for 20 s, 55°C or 60°C for 20 s, and 72°C for 50 s. The annealing temperature for each primer set was described in Table 2. The fluorescent products were detected in the last step of each cycle. A melting curve analysis was performed after amplification to distinguish the targeted PCR products from the non-targeted ones. The melting curve was obtained by slow heating at temperatures from 60 to 95°C at a rate of 0.2°C/s with continuous fluorescence collection. Amplification and detection were performed in 384-well optical plates with an ABI PRISM 7900HT sequence detection system (Applied Biosystems, Foster, CA). Standard curves for the standard bacterial strains in Table 2 were generated by using C_q_ values and the corresponding cell counts, which were determined microscopically with the DAPI staining method as previously described [20]. To determine the target bacterial populations in the fecal samples, 1/20,000, 1/200,000, and 1/2,000,000 portions of the RNA solution were subjected to RT-qPCR. The quantification cycle (C_q_) values in the linear range of the assay were applied to the analytical curve generated in the same experiment to obtain the corresponding bacterial count in each nucleic acid sample; this count was then converted to the count per sample. No PCR inhibition was observed in each assay (data not shown).

### qPCR

The specific primer set for *C*. *coccoides* group (Table 2) was used. Each reaction mixture (10 μl) was composed of 1× PCR buffer (Takara Bio Inc., Shiga, Japan), each deoxynucleoside triphosphate at a concentration of 200 μM, MgCl_2_ solution at a concentration of 2.5 mM, a 1:75,000 dilution of SYBR green I (BioWhittaker Molecular Applications), Takara Taq (Takara Bio Inc.) at a concentration of 0.02 units/μl, TaqStart antibody (Takara Bio Inc.) at a concentration of 5.5 ng/μl and 5 μl template DNA. g-Ccoc-F and g-Ccoc-R primers were added at a concentration of 0.2 μM in accordance with the method used in a previous report [21]. The amplification program consisted of one cycle at 95°C for 5 min, followed by 40 cycles at 94°C for 20 s, 55°C for 20 s, and 72°C for 50 s. The fluorescent products were detected in the last step of each cycle. A melting curve analysis was performed as described in the methods for RT-qPCR. qPCR amplification and detection were performed in 384-well optical plates with an ABI PRISM 7900HT sequence detection system (Applied Biosystems). Standard curves for the standard bacterial strain in Table 2 were generated by using C_q_ values and the corresponding cell counts, which were determined microscopically with the DAPI staining method as previously described [20]. To identify the target bacterial population in the fecal samples, 1/2,000, 1/20,000, and 1/200,000 portions of DNA solution were subjected to qPCR. The C_q_ values in the linear range of the assay were applied to the analytical curve generated in the same experiment to obtain the corresponding bacterial count in each nucleic acid sample; this count was converted to the count per sample. No PCR inhibition was observed in each assay (data not shown).

### Determination of bacterial counts by FISH

FISH analyses with the *C*. *coccoides* group-specific oligonucleotide probe Erec482 (5’-GCTTCTTAGTCARGTACCG-3’) [22] were performed as described previously [23]. Briefly, fresh bacterial cultures were fixed with three volumes of 4% paraformaldehyde at 4°C for 16 h. Then, 10 μl of fixed-cell suspension at the appropriate dilution was smeared on a MAS-coated slide glass (Matsunami Glass Ind., Ltd., Osaka, Japan), which was hybridized with the probe. Observation and acquisition of the fluorescent images were performed with a Leica imaging system (using a Leica DM6000 automatic fluorescent microscope), image-acquisition software QFluoro, and a cooled black-and-white charge-coupled display camera (Leica DFC3500FX) (Leica Microsystems GmbH, Wetzlar, Germany). The fluorescent images obtained were analyzed by using image analysis software (Image-Pro Plus v. 4.5; Media Cybernetics, Inc., Bethesda, MD) to quantify the fluorescent cells in each sample. Microscopic counts were determined for 10 images per sample.

### Specificity check

The specificity of the primer sets was confirmed against the total RNA fractions extracted from 10^5^ cells of each bacterial strain (Table 1) by using RT-qPCR. The amplified signal was considered positive (+) at >10^4^ standard cells, positive/negative (±) at 10^4^ to 10^0^ standard cells, and negative (-) at <10^0^ standard cells. The amplified signal was also defined as negative (-) when the corresponding melting curve had a peak different from that of the standard strain.

### Sequencing of the RT-PCR amplified products

RT-PCR products generated with the primer set s-Casp-F and s-Casp-R were purified by using a HighPure PCR product purification kit (Roche Diagnostics GmbH, Mannheim, Germany) and used for sequence analysis.

### Statistical analyses

The log-transformed bacterial count was used for statistical analyses. Bacterial counts of “not detected” (ND) samples were regarded as half the detection limits of the corresponding primer sets. JMP version 9.0 software (SAS Institute, Cary, NC) was used to conduct the Steel-Dwass test, which is a popular non-parametric method for multiple comparisons, and Fisher’s probability test with the Holm correction was used to compare the ratio among the 3 age groups. For multivariate analyses of the data, a principal component analysis (PCA) was used to visualize the data sets by using the statistical programming language R 2.1.5. A heatmap was created by using the R function “heatmap.” Hierarchical clustering was based on Ward's minimum variance method and the Euclidean distance metric.

## Results

### Comparison of RT-qPCR with FISH and qPCR for quantification of *C*. *coccoides* group populations in human feces

We compared the RT-qPCR method with FISH and qPCR, which are well-established methodology targeting RNA and DNA, respectively, for enumeration of the *C*. *coccoides* group in fecal samples from 8 healthy volunteers. The population levels of *C*. *coccoides* group determined by RT-qPCR using the *C*. *coccoides* group-specific primer set were statistically equivalent to the results obtained by using FISH. The population levels determined by qPCR were significantly higher than those determined by using RT-qPCR (Steel-Dwass test, *P*<0.01) (Table 3).

**Table 3:** Comparison of C. coccoides group population levels in human feces, as demonstrated by RT-qPCR, FISH and qPCR.

Subject	Bacterial counts (log_10_ cells/g feces)		
	RT-qPCR	FISH^a^	qPCR
A	10.3	10.0	10.5
B	10.3	10.1	10.9
C	10.3	10.2	10.9
D	10.6	10.3	10.8
E	10.2	10.0	10.5
F	10.0	9.8	10.7
G	10.4	10.2	10.7
H	10.5	10.3	11.0
AV	10.3^b^	10.1^c^	10.8
SD	0.2	0.2	0.2

a The *C. coccoides* group-specific probe (Erec482: 5'-GCTTCTTAGTCARGTACCG-3') was used.

b Indicates a significant difference between RT-qPCR and qPCR with Steel-Dwass test (*P*<0.01)

c Indicates a significant difference between FISH and qPCR with Steel-Dwass test (*P*<0.01)

### Specificity of the designed primers

The specificity of the newly designed primers was evaluated by RT-qPCR using the total RNA fractions extracted from 10^5^ cells of the 43 strains tested. Each primer set gave positive RT-qPCR results only for the corresponding target bacterial species within the range of 3 C_q_ values (Table 1). The primer sets g-Blau-F/R, s-Csym-F/R, s-Casp-F/R, and s-Rgna-F/R cross-reacted only weakly with some of the non-target *C*. *coccoides*group strains, at negligible levels, having little effect on the specific enumeration of target bacteria. The detection limit of the RT-qPCR system was 10^–1^ cells per reaction; this was equivalent to about 10^5^ cells/g feces.

### Comparison of *C. coccoides* group populations among different age groups

The total count of *C*. *coccoides* group was 10^9.8±0.3^ cells/g feces in young children and 10^10.0±0.5^ cells/g feces in adults (mean±SD), whereas that in the elderly was 10^9.3±0.9^ cells/g feces significantly lower than those in young children and adults (Steel-Dwass test, *P*<0.01) (Table 4). The sum of the bacterial counts obtained by using the 22 newly developed primer sets was 10^9.7±0.3^ cells/g feces (99.4% of the total) in young children, 10^9.8±0.4^ cells/g feces (99.8% of the total) in adults, and 10^9.3±0.8^ cells/g feces (100% of the total) in the elderly. The bacterial counts of the genus *Blautia* were the highest among the 3 subgroups and 19 species in all three generation groups tested, suggesting that genus *Blautia* predominates in human intestinal *C*. *coccoides-*group populations regardless of age. The prevalences of *R*. *gnavus* and *R*. *torques* were also high in all generation groups (84% to 100%). The prevalences of *F*. *saccharivorans* and genus *Dorea* in adults (88% and 88%, respectively) were significantly higher than those in young children (53% and 22%, respectively) and the elderly (16% and 16%, respectively) (Fisher’s exact probability test, *P*<0.01). *Eubacterium ramulus* was detected in 41% of adults but was not detected in the other age groups. In contrast, *C*. *nexile* was detected in most of the young children tested (94%), whereas its detection rates in adults and the elderly were significantly lower at 34% and 50%, respectively (Fisher’s exact probability test, *P*<0.01). The prevalences of *C*. *scindens* and *C*. *hylemonae* in the elderly (94% and 38%, respectively) were significantly higher than those in young children (56% and 9%, respectively) and adults (53% and 3%, respectively) (Fisher’s exact probability test, *P*<0.01). The counts of *Eu*. *ventriosum*, *Eu*. *hallii*, *Eu*. *eligens*, *Eu*. *rectale*, genus *Blautia*, *C*. *symbiosum*, *C*. *asparagiforme*, *A*. *caccae*, *Ro*. *intestinalis*, *Co*. *comes*, *R*. *torques*, and *R*. *lactaris* populations also differed significantly among different age groups (Steel-Dwass test, *P*<0.05).

**Table 4:** Comparison of *C. coccoides* group populations among different groups.

Bacteria	Young children (3.2±0.1 years old, n = 32)		Adults (39±11 years old, n = 32)		Elderly (82±6 years old, n = 32)	
	Bacterial counts^a^ (log_10_ cells/g feces)	Prevalence(%)	Bacterial counts (log_10_ cells/g feces)	Prevalence(%)	Bacterial counts (log_10_ cells/g feces)	Prevalence(%)
*Clostridium coccoides* group	9.8 ± 0.3	100	10.0 ± 0.5^b^	100	9.3 ± 0.9^c^ ^,^ ^d^	100
Sum of 22 subgroups/species	9.7 ± 0.3	100	9.8 ± 0.4	100	9.3 ± 0.8	100
*Eubacterium ventriosum*	7.7 ± 0.5	22	7.9 ± 0.5^b^	59^e^	7.9 ± 0.8^d^	9^g^
*Eubacterium hallii*	7.6 ± 1.1	66	8.1 ± 0.8^b^	88	7.8 ± 1.1^d^	34^f^ ^,^ ^g^
*Coprococcus eutactus*	8.2	3	ND^h^	0	ND	0
*Eubacterium eligens*	8.6 ± 0.8	41	8.3 ± 0.8	41	7.4 ± 1.0^c^ ^,^ ^d^	16
*Eubacterium rectale*	8.4 ± 0.7	47	8.4 ± 0.7	66	6.1 ± 0.6^d^	47
*Eubacterium ramulus*	ND	0	7.5 ± 0.6^b^	41^e^	ND^d^	0^g^
Genus *Blautia*	9.4 ± 0.4	100	9.3 ± 0.5	100	8.9 ± 0.9^c^	100
*Clostridium symbiosum*	7.0 ± 0.6	97	6.7 ± 0.6^b^	53^e^	7.2 ± 0.6^d^	94^g^
*Fuscicatenibacter saccharivorans*	8.4 ± 0.9	53	8.8 ± 0.6^b^	88^e^	7.5 ± 0.7^c^ ^,^ ^d^	16^f^ ^,^ ^g^
*Clostridium asparagiforme*	7.2 ± 0.5	47	6.2 ± 0.4	50	7.4 ± 0.6^c^ ^,^ ^d^	97^f^ ^,^ ^g^
*Clostridium hathewayi*	6.8 ± 0.7	66	6.7 ± 0.7	50	7.0 ± 0.8	72
*Clostridium indolis* subgroup	ND	0	ND	0	5.8	3
*Ruminococcus gnavus*	8.6 ± 0.8	100	8.1 ± 0.9	97	8.3 ± 0.9	91
*Anaerostipes caccae*	7.3 ± 1.1	94	6.8 ± 0.9^b^	59^e^	7.9 ± 1.1^d^	88^g^
*Roseburia intestinalis*	8.1 ± 1.0	47	6.9 ± 0.8	38	7.7 ± 0.8^c^ ^,^ ^d^	9^f^ ^,^ ^g^
*Coprococcus comes*	6.3 ± 0.7	41	6.7 ± 0.6^b^	66	6.1 ± 0.5^d^	38
*Clostridium nexile*	7.5 ± 0.9	94	7.1 ± 0.8^b^	34^e^	7.1 ± 0.8^c^	50^f^
Genus *Dorea*	8.6 ± 0.3	22	8.2 ± 0.6^b^	88^e^	8.0 ± 1.1^d^	16^g^
*Clostridium scindens*	8.2 ± 0.7	56	6.8 ± 0.8	53	8.8 ± 0.6^c^ ^,^ ^d^	94^f^ ^,^ ^g^
*Clostridium hylemonae*	6.9 ± 0.8	9	6.0	3	6.7 ± 0.6^c^ ^,^ ^d^	38^f^ ^,^ ^g^
*Ruminococcus torques*	7.6 ± 1.2	84	6.8 ± 0.8	91	8.7 ± 0.6^c^ ^,^ ^d^	94
*Ruminococcus lactaris*	8.8	3	8.2 ± 0.9^b^	25	6.3 ± 0.9	19

a Data are expressed as the means and standard deviations.

b Indicates a significant difference between young children and adults with Steel-Dwass test (*P*<0.05)

c Indicates a significant difference between young children and elderly with Steel-Dwass test (*P*<0.05)

d Indicates a significant difference between adults and elderly with Steel-Dwass test (*P*<0.05)

e Indicates a significant difference between young children and adults with Fisher's exact probability test after Holm correction (*P*<0.05)

f Indicates a significant difference between young children and elderly with Fisher's exact probability test after Holm correction (*P*<0.05)

g Indicates a significant difference between adults and elderly with Fisher's exact probability test after Holm correction (*P*<0.05)

h Not detected

**Fig 2:**
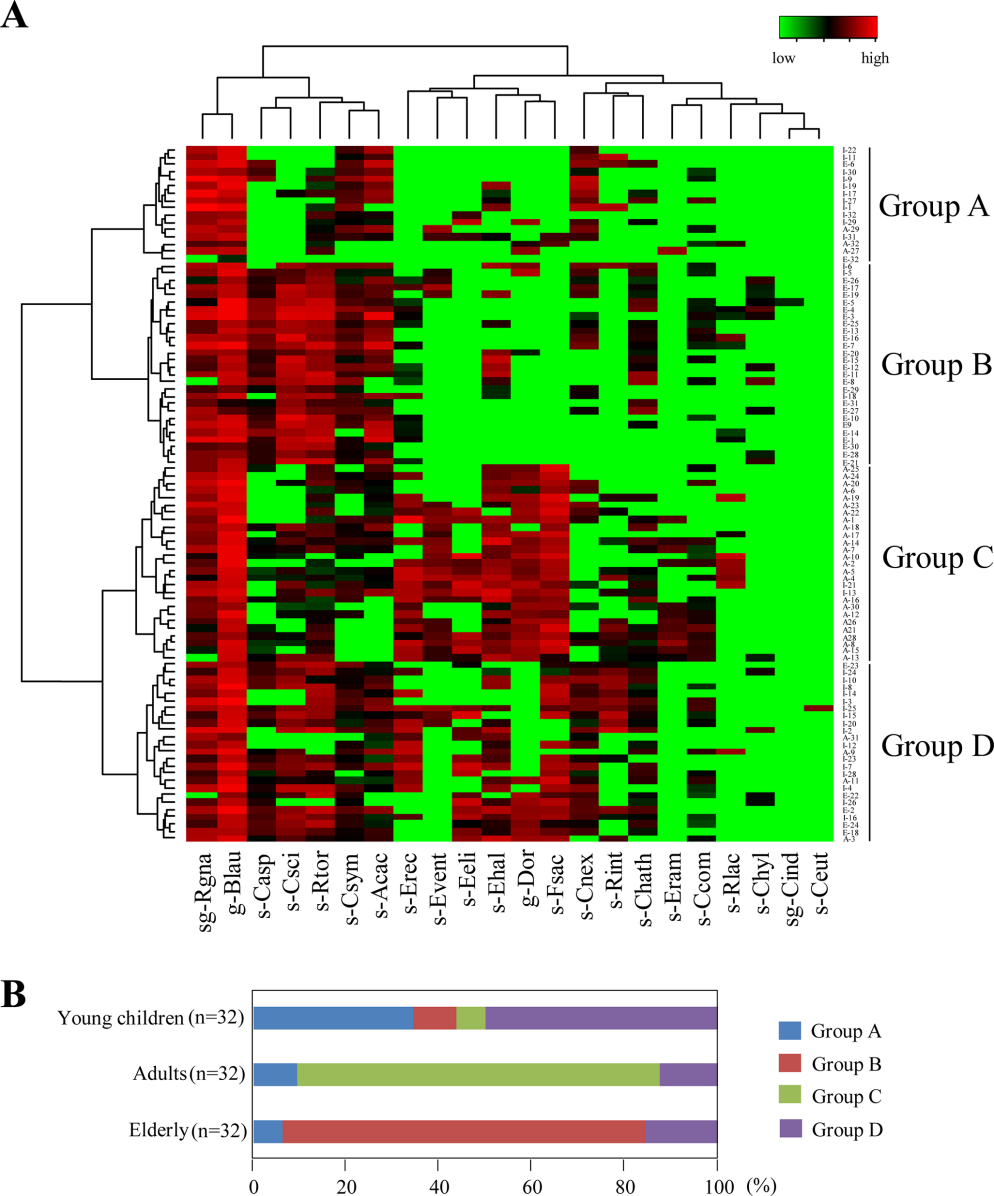
Heatmap analysis of the counts of *C*. *coccoides* group populations enumerated by using 22 primer sets in 32 young children, 32 adults, and 32 elderly. (A) Hierarchical clustering with a heatmap representation based on Ward's minimum variance method and a Euclidean distance metric. Subject IDs of 32 young children (I-1 to I-32), 32 adults (A-1 to A-32), and 32 elderly (E-1 to E32) are shown at the right side of the heatmap. The subjects tested were classified into 4 groups (Groups A to D) by hierarchical clustering. Colors ranging from green to red indicate low to high population levels. (B) Classification of subjects by the hierarchical clustering. Most of the adults (78%) were classified into Group C and most of the elderly (78%) into Group B, Specific clusters were not as apparent in children.

**Fig 3:**
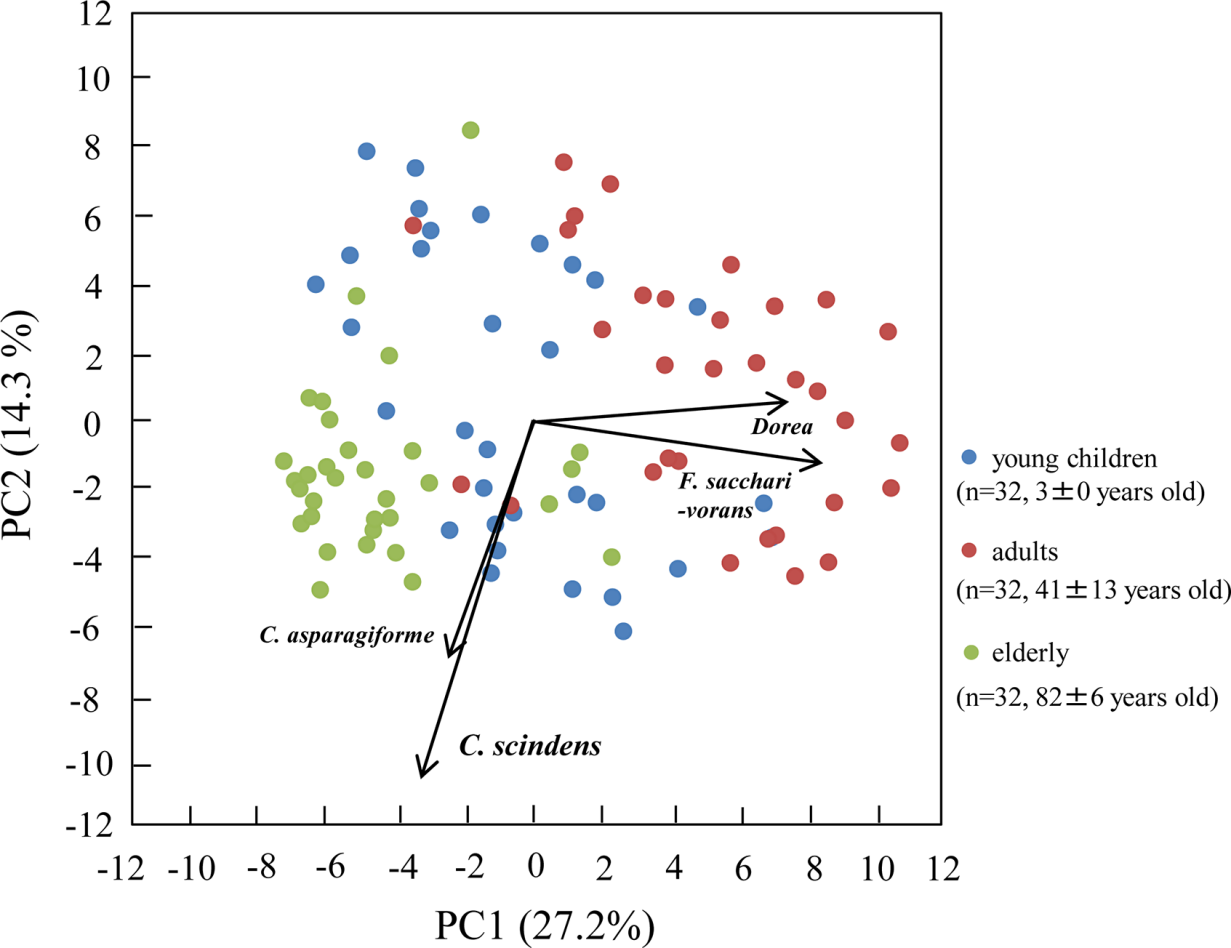
Principal component analysis (PCA) of bacterial counts determined by using 22 primers in 32 young children, 32 adults, and 32 elderly. Blue, red, and green plots show young children, adults and the elderly, respectively. Arrows indicate characteristic vectors of the upper 4 factor loadings.

In accordance with the hierarchical clustering of all the data sets, the 96 subjects were classified into 4 groups (Groups A to D) (Fig 2). Seventy-eight percent of the adults were classified into the same group (Group C), whereas 78% of the elderly belonged to another group (Group B) (Fig 2). Thirty-four percent of the young children were classified into Group A and 50% into Group D. The results of PCA supported our finding of clear differences among the age groups—especially between the plots of adults and those of the elderly along the PC1 axis, which had high degrees of correlation with the counts of *F*. *saccarivorans* and genus *Dorea* (Fig 3). Both the heatmap analysis and the PCA visualized diverse bacterial patterns among individuals (Figs 2 and 3). Taken together, these results indicated that *C*. *coccoides*-group populations clearly differed among the age groups.

## Discussion

Culture-dependent and—independent approaches, including sequence-based methods, have produced large numbers of data sets of dominant populations in the human intestinal microbiota, as well as a catalog of prevalent microbial genes [1,24–28], but few studies have focused on the *C*. *coccoides* group. This seems to be the result of the methodological limits of the culture methods or PCR, i.e. the selection media or specific primer sets at the species level in this group have been insufficient for identification. We initially classified the diverse *C*. *coccoides* group into 3 subgroups and 19 species. We then developed a highly sensitive RT-qPCR system for quantification of the *C*. *coccoides* group by using 22 specific primer sets.

The population levels of *C*. *coccoides* group in the intestine, as determined by using RT-qPCR, were equivalent to those determined by using FISH but significantly lower than those determined by using qPCR (Table 3). This was likely due to the difference in target molecules among these methods: both RT-qPCR and FISH target bacterial RNA molecules, whereas qPCR targets bacterial DNA molecules. RNA molecules can be degraded more rapidly after cell death than can DNA molecules and are produced only by metabolically active cells; they are therefore indicators of bacterial cell viability [29,30]. Although it has been reported that rRNA, unlike mRNA, can persist for extended periods in dead cells [31], several *in vitro* analyses of both anaerobic and aerobic bacterial cultures have shown that the bacterial counts obtained by using RT-qPCR are in good agreement with those obtained by using culture methods even when the cells are dead, suggesting that the counts of viable cells can be quantified accurately by using RT-qPCR targeting rRNA [14,15,17]. Unlike RNA, bacterial DNA cannot be degraded rapidly in dead cells, and this may lead to the mis-quantification of substantial numbers of dead cells along with viable cells by qPCR [14]. Therefore, the differences in the counts between qPCR and RT-qPCR (Table 3) were likely due to overestimation of the bacterial counts by the DNA-targeted method. The slight differences in the bacterial counts obtained by FISH and RT-qPCR (10^0.1^ to 10^0.3^ cells/ g feces) might have been due to technical issues. FISH gives positive or negative signals (i.e. the cell is detected above a certain threshold of rRNA molecules, regardless of how many are present), whereas the RT-qPCR signals are quantitative and are directly related to the number of rRNA molecules present.

The gastrointestinal tract is colonized first by facultative anaerobes immediately after birth, and consumption of oxygen by these bacteria is followed by the growth of strict anaerobes [32]. Previous studies have shown that the *C*. *coccoides* group in the intestines is established at an early age and remains stable with age [33,34]. Our results also support the hypothesis that overall counts of the *C*. *coccoides* group in young children are similar to those in adults. However, the RT-qPCR analysis at the subgroup and species levels demonstrated differences in composition among the different age groups. For example, *Eu*. *ramulus* characterized the intestinal microbiota in adults (Table 4); this bacterium has been reported to grow with quercetin-3-glucoside (isoquercitrin), a flavonoid, as the sole carbon and energy source [35]. Flavonoids are polyphenolic compounds present in foods and beverages of plant origin [36], and the growth of *Eu*. *ramulus* is stimulated by ingestion of flavonoids *in vivo* [37]. Unlike that of *Eu*. *ramulus*, the incidence of *C*. *nexile* was higher in young children (94%) than in adults (34%) (Table 4); thus *C*. *nexile*, which produces antimicrobial substances [38], characterizes the intestinal microbiota of young children. Growth of *C*. *nexile* is inhibited by garlic [39], which is not usually included in the regular meals of younger children. These results suggest that diet affects the composition of the intestinal *C*. *coccoides* group.

Decreases in population levels of the *C*. *coccoides* group are correlated with age-related events such as the development of frailty, hospitalization, antibiotic treatment, and non-steroidal anti-inflammatory therapy [40–43]. Population levels of the intestinal *C*. *coccoides* group in our elderly group were significantly lower than those in young children and adults (Table 4), supporting the data from Japanese, Italian and Finnish studies [44–46]. The lower *C*. *coccoides* group population levels in the elderly could be due to the lower levels of the genus *Blautia* as the predominant population in this group (Table 4). Genus *Blautia* has recently been postulated to be a novel group by its reclassification on the basis of 16S rRNA sequencing [47], and a number of isolates belonging to this genus have recently been identified from human feces by using methods such as FISH, flow cytometry, clone library analysis, and metagenomic analysis [1,6,48]. In a Japanese study, Hayashi *et al*. [6] have shown that *R*. *obeum*, a member of the genus *Blautia*, commonly inhabits the human intestine regardless of age. The genus was found in all the subjects in this study, and the population levels tend to diminish with age (Table 4). Intestinal levels of the genus *Blautia* can change with a number of conditions. For example, intestinal population levels of *Blautia* in children with type 1 diabetes are significantly higher than those in healthy children [49], and there is a significant reduction in the *Blautia* population in the intestines of cirrhotic patients [50]. These results and ours suggest that the reduced population levels of the genus *Blautia* in the elderly may have a substantial effect on health in this age group.

The hierarchical clustering and PCA results showed that the patterns of intestinal microbiota in the elderly were clearly different from those in adults (Figs 2 and 3): the elderly were characterized by high frequencies of *C*. *scindens* and *C*. *asparagiforme* and low frequencies of *F*. *saccharivorans* and genus *Dorea* (Table 4, Fig 3), suggesting that these species might be useful age markers. The function of species that predominate in the elderly is unclear, as is the function of *F*. *saccharivorans*, a novel species only recently isolated from human feces [7]. In contrast, *C*. *scindens* is known to be responsible for bile acid 7α-dehydroxylation; this contributes to the production of secondary bile acids such as deoxycholic acid and lithocholic acid [10], which are associated with increased risk of gallstone disease and colon cancer [51,52]. Interestingly, the prevalence of *C*. *hylemonae*, another species with high bile acid 7α-dehydroxylating activity [10], was significantly higher in the elderly than in adults and young children (Table 4). The abundance of secondary bile acid-producers in the elderly might have harmful impacts on the health of this group. In contrast with the PCA plots of adults and the elderly, the plots of young children showed wide scattering along both the PC1 and the PC2 axis (Fig 3). Moreover, hierarchical clustering showed that the group composition for young children clearly differed from those for the adults and elderly (Fig 2). Although it has been reported that the population levels of major anaerobes in children aged 3 years are similar to those in adults [34], our results indicated that the composition of the *C*. *coccoides* group was not yet stable at this age. However, because the numbers of subjects and age groups were limited in our study, large numbers of subjects covering wider age groups need to be evaluated by using the RT-qPCR system to examine dynamic shifts over entire lifetimes.

In conclusion, our 16S rRNA-targeted RT-qPCR system for the *C*. *coccoides* group gave accurate information on the composition of the major intestinal anaerobes. We demonstrated a dramatic change in *C*. *coccoides*-group populations throughout life: those in young children are unstable and highly diverse among individuals, and those in adults are clearly different from those in the elderly. These results suggest that a number of factors affect the composition of *C*. *coccoides*-group populations; at the same time, the diversity of these populations likely helps to maintain intestinal homeostasis in the different age groups.
