# Correction: Diversity of Intestinal *Clostridium coccoides* Group in the Japanese Population, as Demonstrated by Reverse Transcription-Quantitative PCR

**DOI:** 10.1371/journal.pone.0152753

**Published:** 2016-03-28

**Authors:** Takashi Kurakawa, Kiyohito Ogata, Kazunori Matsuda, Hirokazu Tsuji, Hiroyuki Kubota, Toshihiko Takada, Yukiko Kado, Takashi Asahara, Takuya Takahashi, Koji Nomoto

There are multiple errors in the values for product size in Table 2, “Primer information.” Please see the corrected Table 2 here.

**Table 2:** Primer information.

group/species	Standard Strain	Strain No.	Primer name	Sequence (5'-3')	Anealing Temp (°C)	Product size (bp)
*Clostridium coccoides* group	*Blautia producta*	JCM 1471^T^	g-Ccoc-F	AAATGACGGTACCTGACTAA	55	440
			g-Ccoc-R	CTTTGAGTTTCATTCTTGCGAA		
*Eubacterium ventriosum*	*Eubacterium ventriosum*	ATCC 27560^T^	s-Event-F	GTCGGGGGACAATAGTTCC	55	451
			s-Event-R	ATTTGCTTACCCTCACGGGG		
*Eubacterium hallii*	*Eubacterium hallii*	DSM 3353^T^	s-Ehal-F	GTGTCGGGGCCGTATAGG	55	435
			s-Ehal-R	GTTCGCCTCACTCTGTGAC		
*Coprococcus eutactus*	*Coprococcus eutactus*	ATCC 27759^T^	s-Ceut-F	CTGGAGCTTGCTCCGGCCGATTT	55	656
			s-Ceut-R	GTCAGTAGCAGTCCAGTAAGT		
*Eubacterium eligens*	*Eubacterium eligens*	DSM 3376^T^	s-Eeli-F	TGTCGGGGCCCATAAGGG	55	189
			s-Eeli-R	CATTACTGTCCGGTCAGTG		
*Eubacterium rectale*	*Eubacterium rectale*	ATCC 33656^T^	s-Erec-F	TTCTGACCGGTACTTAACCGTACC	55	281
			s-Erec-R	TTTGCTCGGCTTCACAGCTTT		
*Eubacterium ramulus*	*Eubacterium ramulus*	ATCC 29099^T^	s-Eram-F	GAGCGTAGGCGGTCCTGC	55	452
			s-Eram-R	GGGAAAACACATTACATGTTCTG		
Genus *Blautia*	*Blautia producta*	JCM 1471^T^	g-Blau-F	GTGAAGGAAGAAGTATCTCGG	55	559
			g-Blau-R	TTGGTAAGGTTCTTCGCGTT		
*Clostridium symbiosum*	*Clostridium symbiosum*	JCM 1297^T^	s-Csym-F	TAAGCGCACAGTATTGCATGATA	55	816
			s-Csym-R	CGTTACTCCCCCGTCGAG		
*Fusicatenibacter saccharivorans*	*Fusicatenibacter saccharivorans*	JCM 18507^T^	s-Fsac-F	CTGCATTGGAAACTGTCTGG	55	388
			s-Fsac-R	CGTTACGGGCCGGTCATC		
*Clostridium asparagiforme*	*Clostridium asparagiforme*	DSM 15981^T^	s-Casp-F	GTTTTCGGATGGATTCTAGATG	55	568
			s-Casp-R	CTCCTGCACTCTAGCTTGA		
*Clostridium hathewayi*	*Clostridium hathewayi*	DSM 13479^T^	s-Chath-F	CTTGACATCCCACTGAAAACAC	55	162
			s-Chath-R	AGAGTGCCCGACTCTACTC		
*Clostridium indolis* subgroup	*Clostridium indolis*	JCM 1380^T^	sg-Cind-F	ACCAAGTCTTGACATCGGAATGA	55	298
			sg-Cind-R	TTGCTCCAGATCGCTCCTT		
*Ruminococcus gnavus*	*Ruminococcus gnavus*	ATCC 29149^T^	s-Rgna-F	CTTGCTGGACGATGACTGAC	55	288
			s-Rgna-R	CTCCGATTAAAGAGCGGTCAGA		
*Anaerostipes caccae*	*Anaerostipes caccae*	DSM 14662^T^	s-Acac-F	GTTTTCGGATGGATTTCCTATAT	55	143
			s-Acac-R	CTTTTCACACTGAATCATGCGATT		
*Roseburia intestinalis*	*Roseburia intestinalis*	DSM 14610^T^	s-Rint-F	GCACAGGGTCGCATGACCT	60	818
			s-Rint-R	AACACATTACATGTTCTGTCATC		
*Coprococcus comes*	*Coprococcus comes*	ATCC 27758^T^	s-Ccom-F	GTGACCGGCGTGTAATGACG	55	150
			s-Ccom-R	CAGAGTGCCCATCCGAATTG		
*Clostridium nexile*	*Clostridium nexile*	ATCC 27757^T^	s-Cnex-F	GGATTTCTTCGGATTGAAGTTTTT	55	517
			s-Cnex-R	TTTCACATCAGACTTACACAAC		
Genus *Dorea*	*Dorea formicigenerans*	DSM 3992^T^	g-Dor-F	GCAGCTAACGCAATAAGCAG	55	165
			g-Dor-R	CTTCCATTACGAAGCGGTC		
*Clostridium scindens*	*Clostridium scindens*	JCM 6567^T^	s-Csci-F	GCATTTGGAACTGCGTGG	55	387
			s-Csci-R	CGTTACGCGCTTTGGCATCG		
*Clostridium hylemonae*	*Clostridium hylemonae*	DSM 15053^T^	s-Chyl-F	AAGAGATTAGCTTGCTAAGATCAG	55	145
			s-Chyl-R	TCTACCATGCGGTACTGAGGT		
*Ruminococcus torques*	*Ruminococcus torques*	ATCC 17756^T^	s-Rtor-F	CGAAGCACTTTGCTTAGA	55	528
			s-Rtor-R	ACATCAGACTTGCCCATC		
*Ruminococcus lactaris*	*Ruminococcus lactaris*	ATCC 19176^T^	s-Rlac-F	GGGAGCGTAGACGGAGCA	55	452
			s-Rlac-R	AAGCAGACATTACTCTGCCG		
